# Supplementary material for: The Impact of Hydroxychloroquine on Primary Feto-Placental Endothelial Cells from Healthy and Early-Onset Preeclamptic Placentas
Source: Int J Mol Sci. 2023 Jun 30;24(13):10934. doi: 10.3390/ijms241310934 (PMC10341411; doi:10.3390/ijms241310934)
Supplement: Supplementary file 1 [file ijms-24-10934-s001.zip › ijms-2454564-supplementary.pdf]

Supplementary Materials

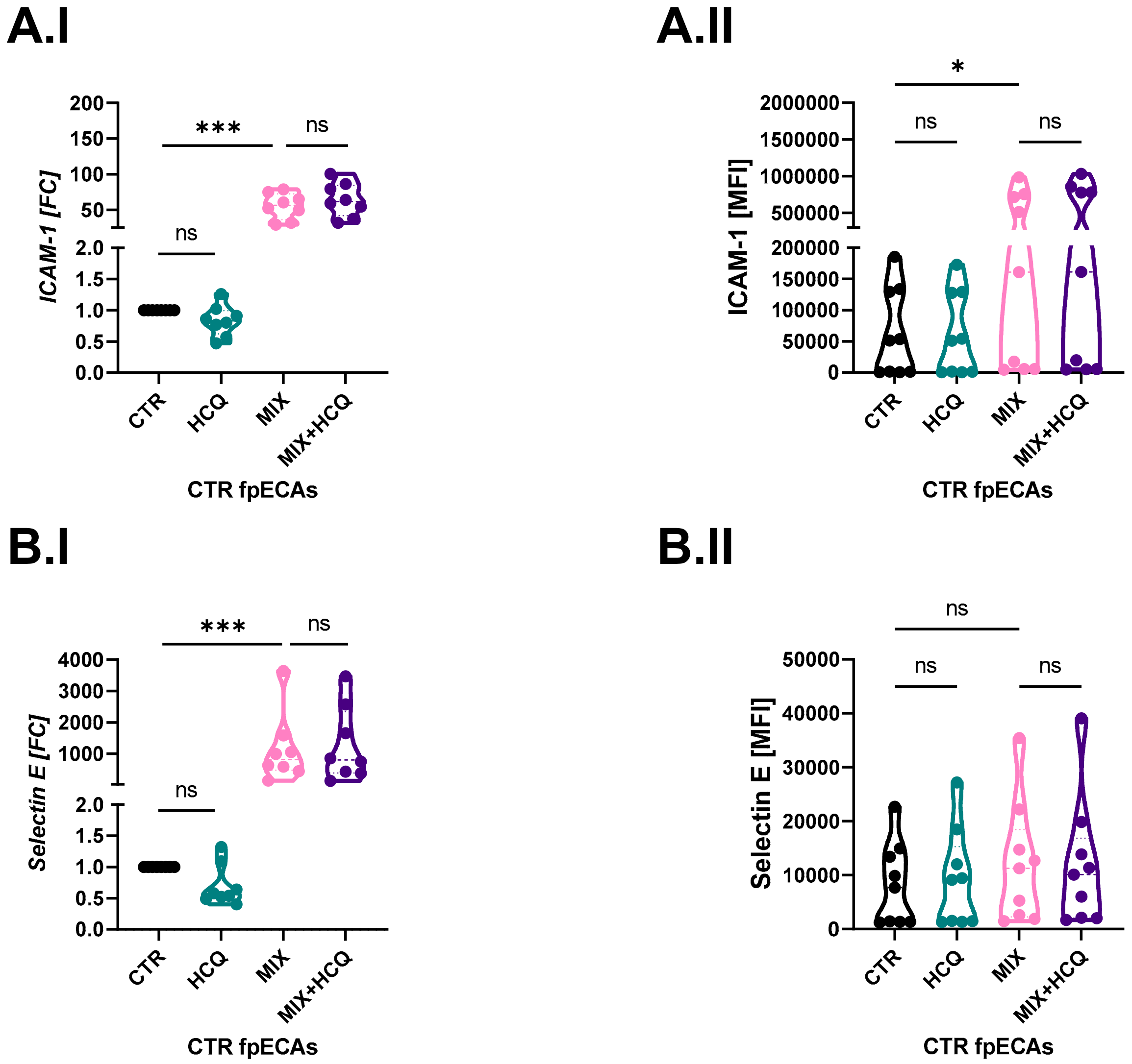

Figure S1 HCQ effect on basal and cytokine induced levels of ICAM-1 (**A**) and SELE (**B**). In case of ICAM-1 MIX significantly increased both mRNA (**A.I**) and membrane presence (**A.II**); *SELE* transcription (**B.I**) was affected only by MIX. Although, increasing trend was present on protein level of SELE (**B.II**), it was not significant. HCQ did not have any effect either alone or in combination with MIX on any level of ICAM-1 or SELE. N = 8 – 9. Repeated measurements (RM) one-way ANOVA with Sidak's post-hoc test was used to determine the significance. *** $p \leq 0.001$, ns= not significant. CTR, control; HCQ, hydroxychloroquine, MIX, cytokine mix; fpECA, feto-placental endothelial cells isolated from arteries; ICAM-1, intercellular adhesion molecule 1; SELE, selectin E.

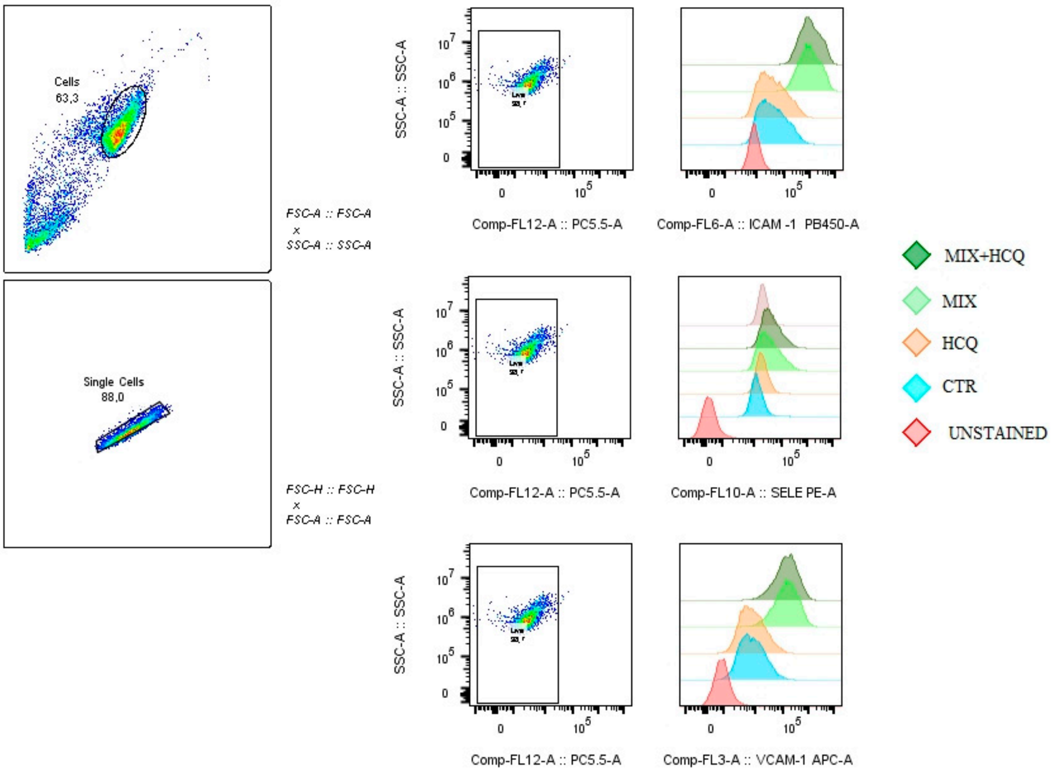

Figure S2 Gating strategy - The cells were initially segregated based on their size using area forward and side scatter measurements (FSC-A and SSC-A). Then, doublet discrimination was carried out by plotting the height of FSC (FCS-H) against the area of FSC (FCS-A). To account for surface protein expression, an additional gating step was applied to separate 7-AAD positive and negative cells, and only the 7-AAD negative population was considered. Finally, ICAM-1, VCAM-1 and SELE positive and negative cell populations were represented in a histogram in each condition with unstained control. VCAM-1, vascular cell adhesion protein 1; SELE, Selectin E.

Table S1 List of primer assays used for the RT-qPCR analysis

**Target**	**Assay**	**Supplier**
18S	Forward (5′-3′) CTACCACATCCAAGGAAGCA<br>Reverse (5′-3′) TTTTTCGTCACTACCTCCCCG	Sigma- Aldrich
RPL30	Hs_RPL30_1_SG QuantiTect Primer Assay (QT00056651)	Quiagen
HPRT1	Hs_HPRT1_1_SG QuantiTect Primer Assay (QT00059066)	Quiagen
IL-6 R1	Hs_IL6R_1_SG QuantiTect Primer Assay (QT00023660)	Quiagen
TNF-α R1	Hs_TNFRSF1A_1_SG QuantiTect Primer Assay (QT00216993)	Quiagen
IL-1β R1	Hs_IL1R1_1_SG QuantiTect Primer Assay (QT00081263)	Quiagen
CXCL8	Hs_CXCL8_1_SG QuantiTect Primer Assay (QT00000322)	Quiagen
ICAM-1	Hs_ICAM1_1_SG QuantiTect Primer Assay (QT00074900)	Quiagen
VCAM-1	Hs_VCAM1_1_SG QuantiTect Primer Assay (QT00018347)	Quiagen
SELE	Hs_SELE_1_SG QuantiTect Primer Assay (QT00015358).	Quiagen

RPL30, Ribosomal Protein L30; 18S, 18 Ribosomal RNA; HPRT1, Hypoxanthine phosphoribosyl transferase 1, HPRT1; IL-6 R1, Interleukin 6 receptor; IL-1β R1, Interleukin 1β receptor 1; TNF-*α* R1, Tumor Necrosis Factor *α* receptor 1; CXCL8, Interleukin 8.
